# Supplementary material for: Inverse-design magnonic devices
Source: Nat Commun. 2021 May 11;12:2636. doi: 10.1038/s41467-021-22897-4 (PMC8113576; doi:10.1038/s41467-021-22897-4)
Supplement: Supplementary file 1 — Supplementary information [file 41467_2021_22897_MOESM1_ESM.pdf]

Supplementary information
Inverse-design magnonic devices

Qi Wang[1], *Andrii V. Chumak*[1], *Philipp Pirro*[2]

[1] *Faculty of Physics, University of Vienna, Boltzmanngasse 5, A-1090 Vienna, Austria*

[2] *Fachbereich Physik and Landesforschungszentrum OPTIMAS, Technische Universität Kaiserslautern, D-67663 Kaiserslautern, Germany*

In the supplementary information, the ten times smaller magnonic demultiplexer is designed using the same algorithm and its functionality is discussed in Section 1. In Section 2, the situation of reversed spin-wave flow direction in the magnonic demultiplexer is discussed. The influence of simulation cell size (Section 3), possible over-etch during the fabrication process (Section 4), and temperature (Section 5) are discussed. In Section 6, the nonlinear shift of the dispersion curves in the nanoscale waveguide is shown. In Section 7, the potential influences of the nonlinearity and possible artifacts in the ground state of the single-layer simulation on the nonreciprocity of the circulator are evaluated. All these additional simulations suggest that our device is robust and manufacturable. In the last section, an additional way to calculate the spectra of the magnonic demultiplexer is discussed.

1. Magnonic demultiplexer with exchange spin waves of ultrashort wavelength

In the main text, we chose a large design region and elements as well as long wavelengths of spin waves due to the current nanofabrication limitations and the reduced excitation efficiency for short wavelengths of spin waves. In order to check the capability of the presented method for small size and short wavelength, we scale down the structure size by 10 times. Figure S1a shows the sketch of the scaled magnonic demultiplexer, which similarly has one input and two output waveguides with width down to 30 nm and a 100×100 nm^2 design region. This design region has been divided into 100 elements, each with a size of 10×10 nm^2. The thickness of the YIG is reduced to 20 nm and the cell size of simulations is decreased to 5 nm. The same external field 200 mT is applied along the out-of-plane direction. The functionality of the scaled demultiplexer is shown in Fig. S1b, in which the spin wave of the frequency f_1 = 16 GHz (wavelength λ = 70 nm) is guided into the first output, whereas the signal of frequency f_2 = 18 GHz (λ = 60 nm) is transmitted to the second output. The crosstalk between the two outputs is less than 5 %. These additional simulations prove that the same inverse design method could be used for smaller structures and shorter wavelengths. The whole size of the

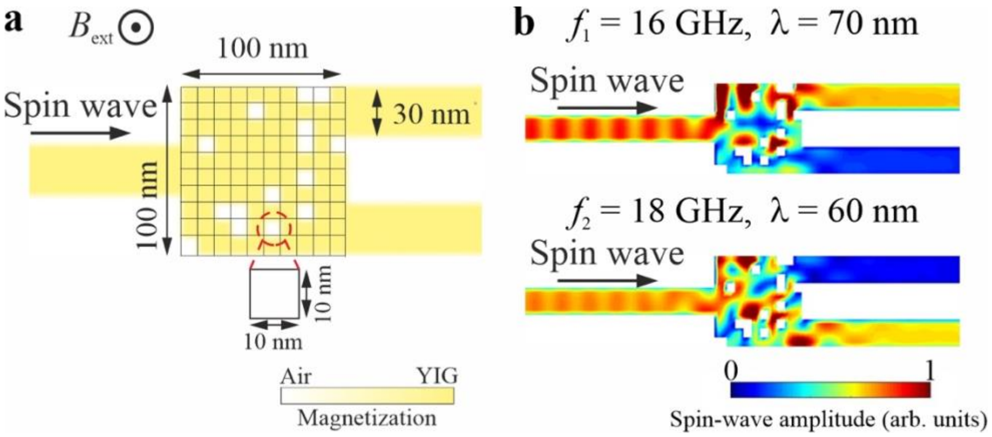

Fig. S1 **a**, The sketch of the scaled magnonic demultiplexer consists of 30 nm wide input and output waveguides, and a 100×100 nm^2 design region, which has been divided into 100 elements each with a size of 10×10 nm^2. The thickness of the YIG is 20 nm. **b**, The normalized spin-wave amplitude for frequencies of 16 GHz and 18 GHz.

scaled demultiplexer is around 200×100 nm^2, which shows another advantage of spin wave, i.e., its short wavelength.

2. Reversed spin-wave flow direction in the magnonic demultiplexer

In the main text, we show that the demultiplexer could efficiently separate the spin waves depending on their frequencies. If the system is perfect reciprocal, one could obtain the multiplexer, which combines different frequencies of spin waves from two inputs into one output waveguide, just by reversing the spin-wave flow direction. Figure S2 shows the spin-

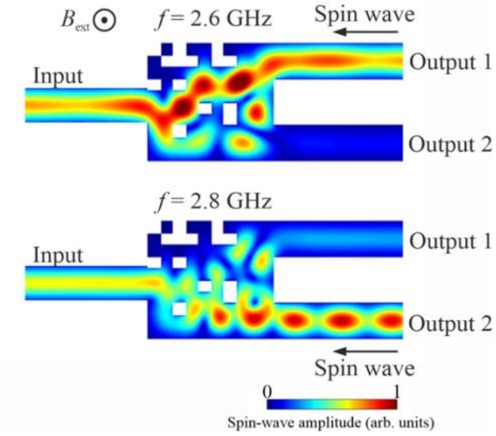

Fig. S2 The simulated spin-wave amplitude of the magnonic demultiplexer with reversed direction of spin-wave flow.

wave amplitude of the demultiplexer with reversed directions of spin-wave flow. For the spin-wave frequency of 2.6 GHz, the device is reciprocal, and all the energy of the spin wave from output 1 is guided into the input and the interference pattern in the design region is similar to

Fig. 3a in the main text. However, for the case of 2.8 GHz, a part of the spin wave from output 2 is guided to output 1 and the interference pattern in the design region is also slightly different comparing Fig. 3a in the main text. This can be explained by the weak nonreciprocity of Forward Volume Spin Waves in a nanoscale waveguide, which has been discussed in the main text.

3. Influence of simulation cell size

Choosing an appropriate resolution is essential for inverse design methods since this can shorten the optimization time without sacrificing precision. In the main text, the simulation cell size was set as 20 nm, which is slightly larger than the exchange length of YIG (~16 nm), but it is much shorter than the minimum studied wavelength (1 μm). Here, we perform additional simulations with smaller cell sizes of 10 nm and studied the influence on the characteristics of the magnonic demultiplexer. The simulated spin-wave amplitude distribution with 10 nm cell size is shown in Fig. S3a, which exhibits minor changes compared to the results for a 20 nm cell size shown in Fig. 3a in the main text. Figure S3b summarized the transmission spectra with different cell sizes. The dashed lines show the results of 10 nm cell size, which slightly shift to higher frequencies. The negligible difference between the two transmissions reveals that 20 nm cell size meets our needs.

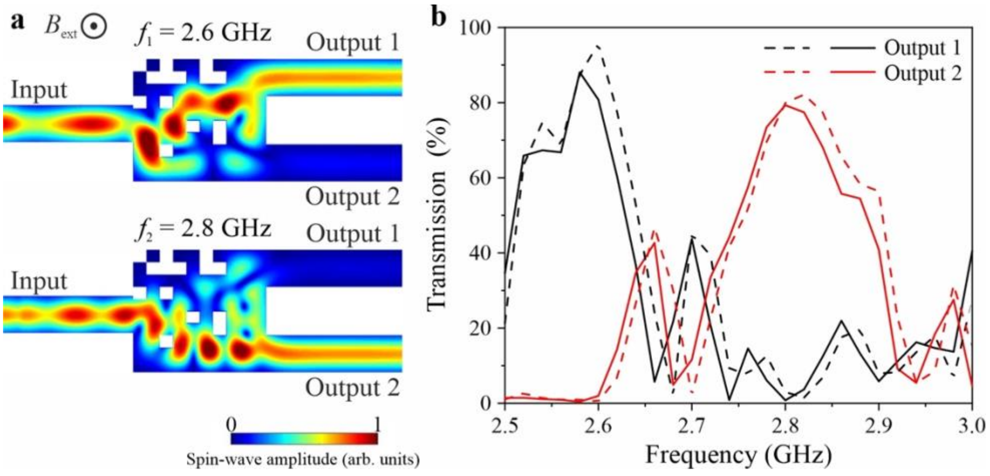

Fig. S3 **a**, The simulated spin-wave amplitude distribution of different spin-wave frequencies with 10 nm cell size. **b**, The transmission as a function of frequency for different cell sizes (dashed lines: 10 nm cell size, solid lines: 20 nm cell size).

4. Fabrication robustness of the magnonic demultiplexer

In this section, we would like to discuss the influence of a potential over-etch, i.e., an unintended increase of the size of the holes which might result from inaccuracies in the

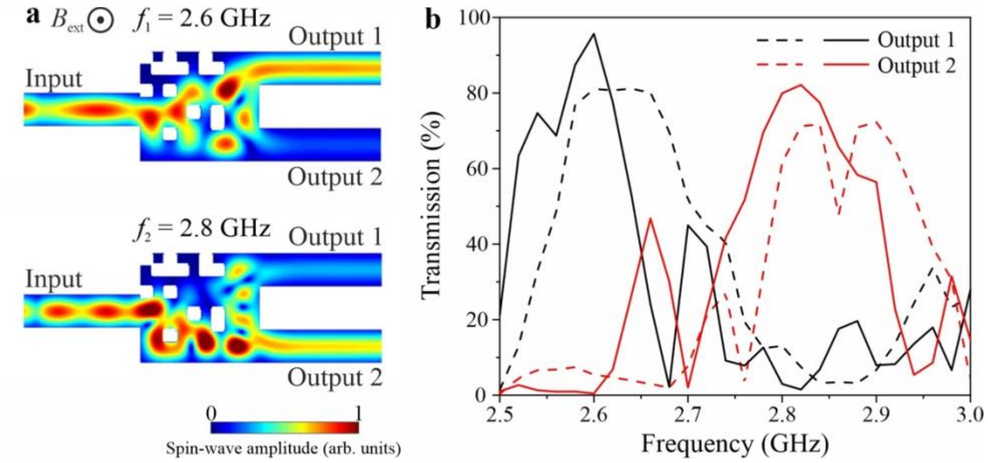

Fig. S4 **a**, The simulated spin-wave amplitude distribution of different spin-wave frequencies with 10 nm over-etched holes. **b**, The transmission as a function of the frequency for the optimized design (solid lines) and the 10 nm over-etch structure (dashed lines).

fabrication process, on the characteristics of the magnonic demultiplexer. Additional simulations were performed with 10 nm over-etched holes on both sizes. The size of the holes is thus 120×120 nm^2 with round corners as shown in Fig. S4a. The area is 40 % larger than the design size (100×100 nm^2). Figure S4b summarized the transmission spectra for different sizes of holes. Only a small difference in the operational characteristics of the demultiplexer was found: The transmission of both frequencies is greater than 60 % and the crosstalk is smaller than 13 % despite up to 40 % area change. Further improvement can be achieved if the details of the fabrication process are included into the optimization algorithm (e.g., direct optimization including the over-etching etc.).

5. Temperature robustness of the magnonic demultiplexer

In the main text, all the simulations were performed without taking into account temperature. Here, additional simulations were performed at an effective temperature of 300 K using the embedded package in Mumax[3] to explore the influence of temperature on the characteristics of the magnonic demultiplexer. Figure S5a and b show that the difference between these two temperatures is neglectable: The transmission and crosstalk of frequency 2.6 GHz are almost

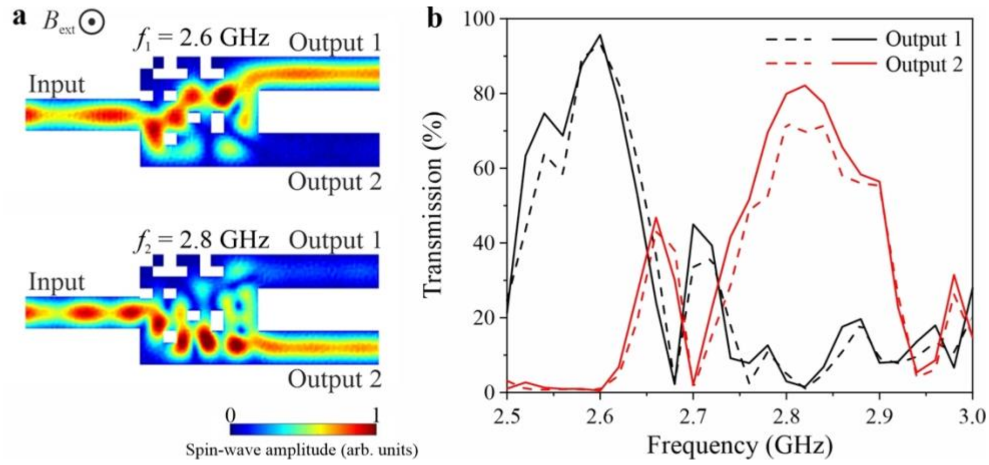

Fig. S5 **a**, The simulated spin-wave amplitude distribution of different spin-wave frequencies at an effective temperature of 300 K. **b**, The transmission as a function of frequency for the temperature of 0 K (solid lines) and 300 K (dashed lines).

identical. The transmission of frequency 2.8 GHz slightly decreases from 80 % to 72 % and the crosstalk is nearly the same.

6. Nonlinear dispersion shift

In this section, we simulate the dispersion curves in a 300 nm wide straight waveguide with a fixed frequency of 2.6 GHz for different excitation fields. Figure S6 shows that the wavenumber of the spin wave is decreased from k = 3.3 rad/μm at b = 0.5 mT to k = 2.8 rad/μm at b = 2.0 mT due to the up-shift of the dispersion curve. This shift is the physical origin of the nonlinear switching shown in the main text.

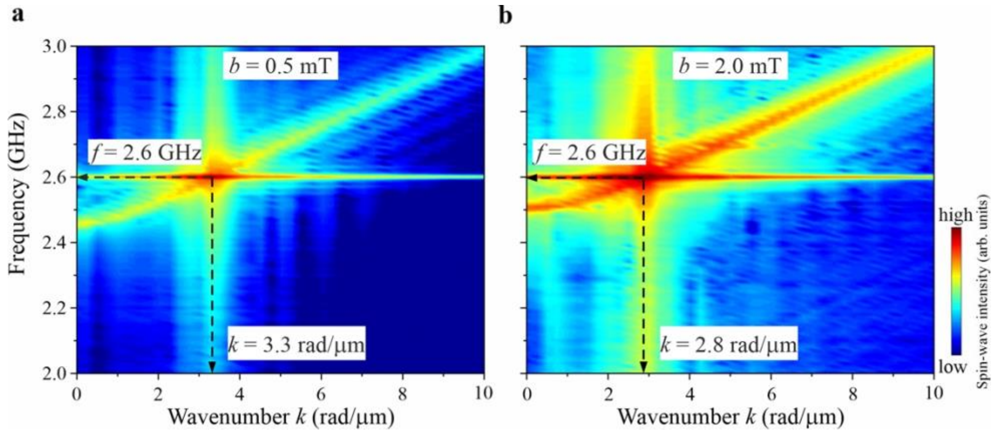

Fig. S6 Simulated spin-wave dispersion curves of fixed frequency of 2.6 GHz in a straight waveguide with (**a**) low excitation field b = 0.5 mT and (b) high excitation filed b = 2.0 mT.

7. Influence of nonlinearity and ground state on nonreciprocity

In the main text, the physics of the nonreciprocal magnonic circulator has been discussed, which is caused by the intrinsic nonreciprocity of the forward volume spin waves in the nanoscaled structure. Here, we evaluate other potential influences on the nonreciprocity, i.e., nonlinearity and possible artifacts in the ground state which might occur in the single-layer simulations. Additional simulations with five times higher damping ($\alpha = 1\times10^{-3}$) are used to evaluate the influence of the nonlinearity on the nonreciprocity. Single-layer simulations are performed in the main text, in which only one layer along the thickness is taken into account, i.e., the profile of the spin wave along the thickness is uniform. This approximation is valid for the studied thickness of 100 nm, what has also been discussed in our previous paper [1].

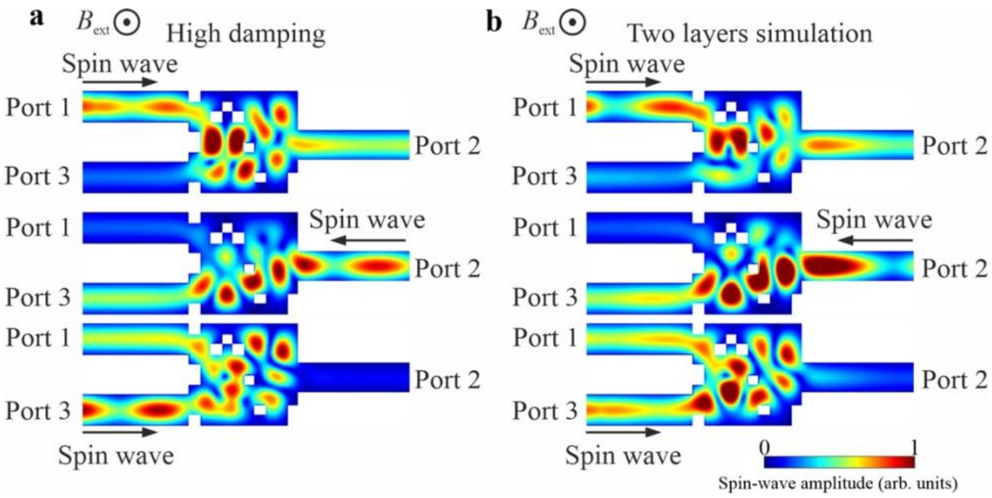

Fig. S7 The normalized spin-wave amplitude in magnonic circulator with (**a**) high damping (1×10^{-3}) and (**b**) two layers simulation along thickness direction.

In order to double-check the influence of the single-layer simulation on the nonreciprocity, additional simulations with two layers along the thickness are performed. Figure S7a and b show the results of the high damping and two layers simulations. No significant change is observed compared to Fig. 5a in the main text, which indicates that the influences of nonlinearity and single-layer simulation can be ignored. The high damping simulations suggest that the circulator can also be fabricated using high damping magnetic metals, which are more compatible with complementary metal-oxide-semiconductor technology.

8. Operational characteristics in the case of the applied sinc pulse

A sinc pulse was widely used to excite a wide range of spin-wave frequencies and then calculate the spin-wave spectra/dispersion curves in single waveguides or magnonic crystals in previous

works. Nevertheless, we should emphasize that in the case of the sinc pulse, the device operates in a pulse regime (namely with many waves of different frequencies and nonstationary amplitudes simultaneously) as opposed to the originally investigated single-frequency continuous-wave (CW) regime.

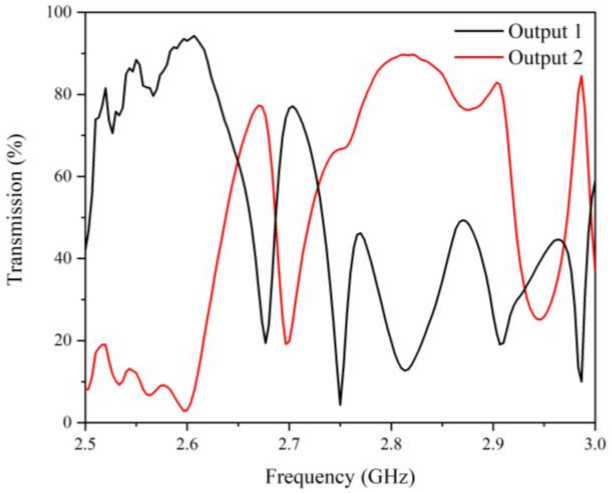

Figure S8. The spin-wave power of different output waveguides as a function of frequency simulated using sinc function excitation.

Here, we demonstrate the simulation of the operational characteristics of the magnonic demultiplexer operating in the pulsed regime. The obtained spin-wave spectra at different outputs are shown in Fig. S8. The result is very similar to the one presented in the original manuscript (Fig. 3b). However, the minimum transmission power is a little bit higher (~3% at 2.6 GHz, ~19% at 2.8 GHz) than what we obtained in the CW regime (~0.5 % at 2.6 GHz, ~3% at 2.8 GHz). The difference can be explained by the following reason: The functionality of the demultiplexer is mainly provided by the wave interference, and the device is optimized for the CW regime when all waves have constant amplitudes. Thus, if to apply a CW signal to the device, an "transition regime" exists before the system reaches a stable interference pattern.

Figure S9 shows the dynamic magnetization (M_z) as a function of simulation time. Three distinct regions are marked in the figure: delay time region, transition region and the region of dynamic equilibrium. In our manuscript, we only perform the FFT in the dynamic equilibrium region which excludes the contribution of the transition region. However, the contribution of the transition region is included in the method of the sinc function excitation and cannot be separated.

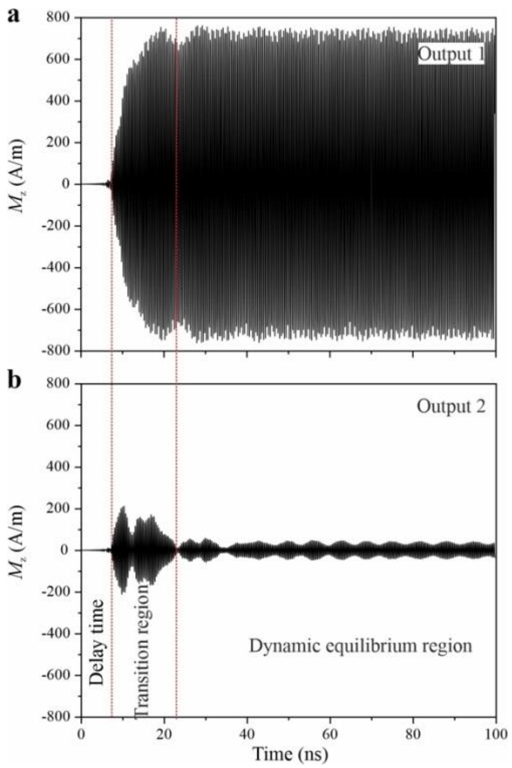

Figure S9. The dynamic magnetization (M_z) as a function of simulation time at different outputs of demultiplexer for operation frequency of 2.6 GHz. Three different regions marked.

The method with sinc pulse excitation is much faster and could be used for the estimation of the operating frequencies of the designed devices. An optimized method would therefore start with a sinc function to obtain a first characterization of the spectra at the outputs and to optimize the frequency separation. Further improvement can then be obtained by testing the relevant frequencies in the dynamic equilibrium using sinusoidal excitation. Another point is that the delay time and the duration of the transition region depend on the group velocity of the spin waves and the size of the devices. It can be further decreased by using high-speed exchange waves or decreasing the size of the devices. Therefore, we expect that the difference between the two methods will reduce further when the size of the device is scaled down and ultrafast exchange spin waves are applied. Finally, a separate optimization of the device for the operations with short (rectangular) pulses is also possible using the presented inverse-design magnonic approach.

Supplementary References:

1. Wang, Q. et al. Spin pinning and spin-wave dispersion in nanoscopic ferromagnetic waveguides. *Phys. Rev. Lett.* **122**, 247202 (2019)
