# Supplementary figures and images for: Inverse-design magnonic devices
Source: Nat Commun. 2021 May 11;12:2636. doi: 10.1038/s41467-021-22897-4 (PMC8113576; doi:10.1038/s41467-021-22897-4)

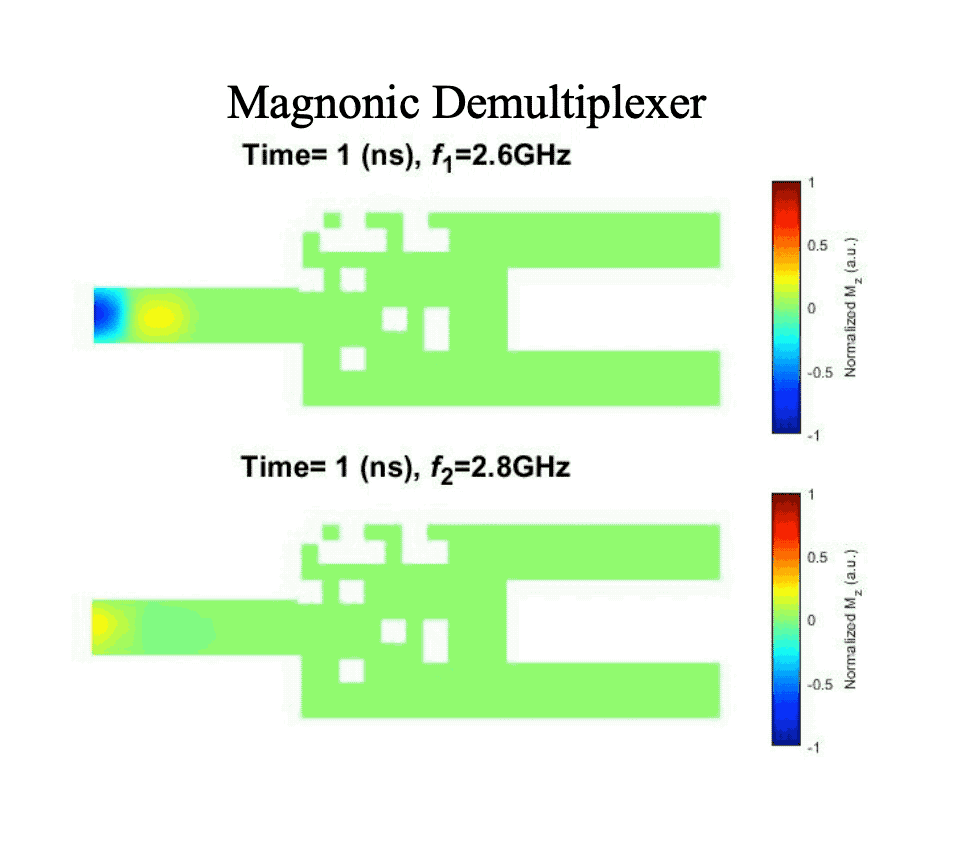

Supplement: Supplementary file 2 — Supplementary Move 1 - Magnonic demultiplexer [file 41467_2021_22897_MOESM2_ESM.gif]

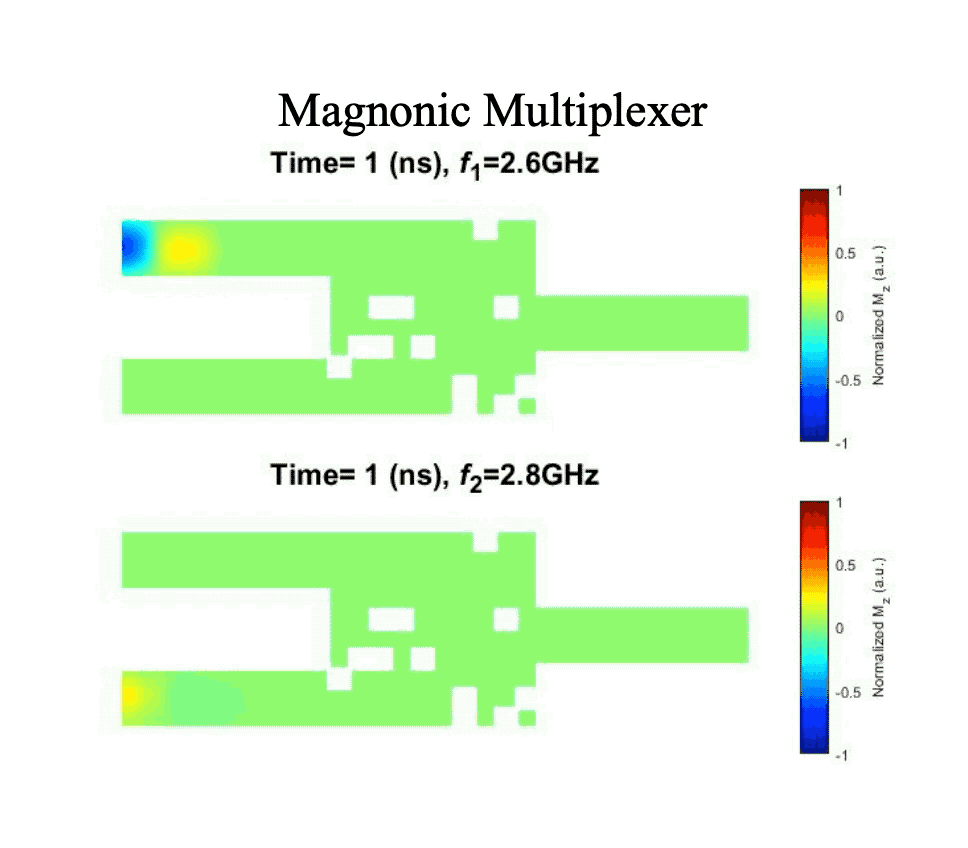

Supplement: Supplementary file 3 — Supplementary Move 2 - Magnonic multiplexer [file 41467_2021_22897_MOESM3_ESM.gif]

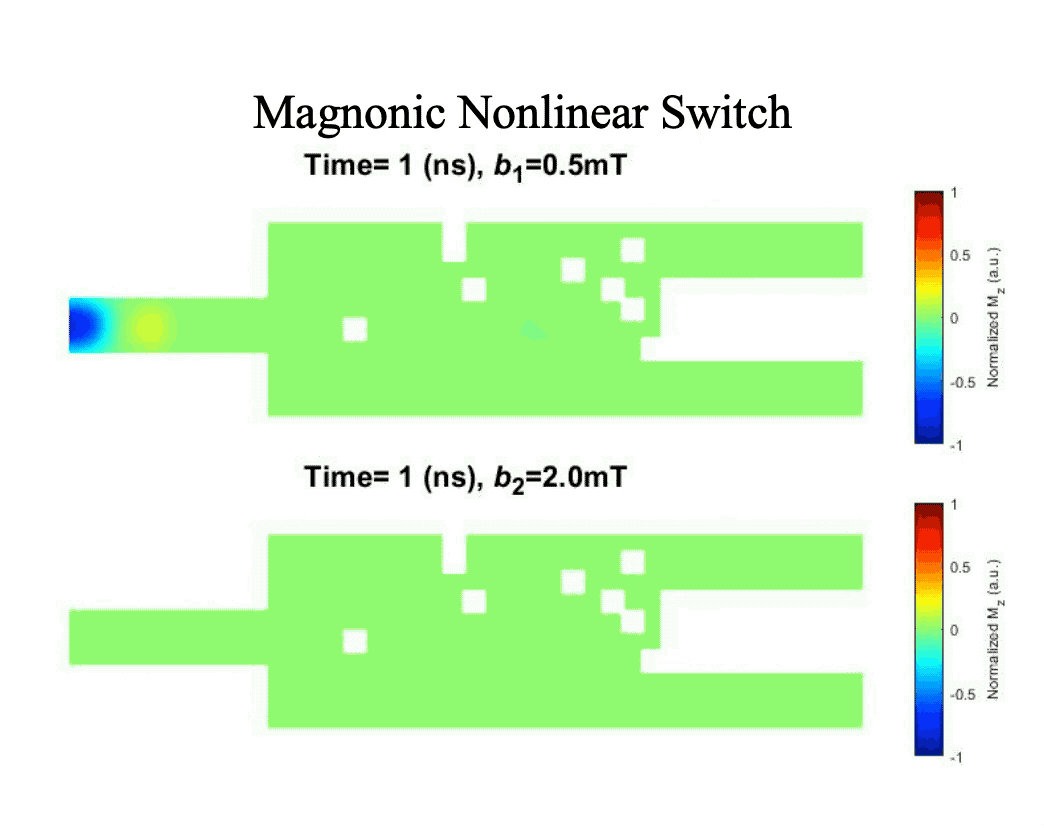

Supplement: Supplementary file 4 — Supplementary Move 3 - Magnonic nonlinear switch [file 41467_2021_22897_MOESM4_ESM.gif]

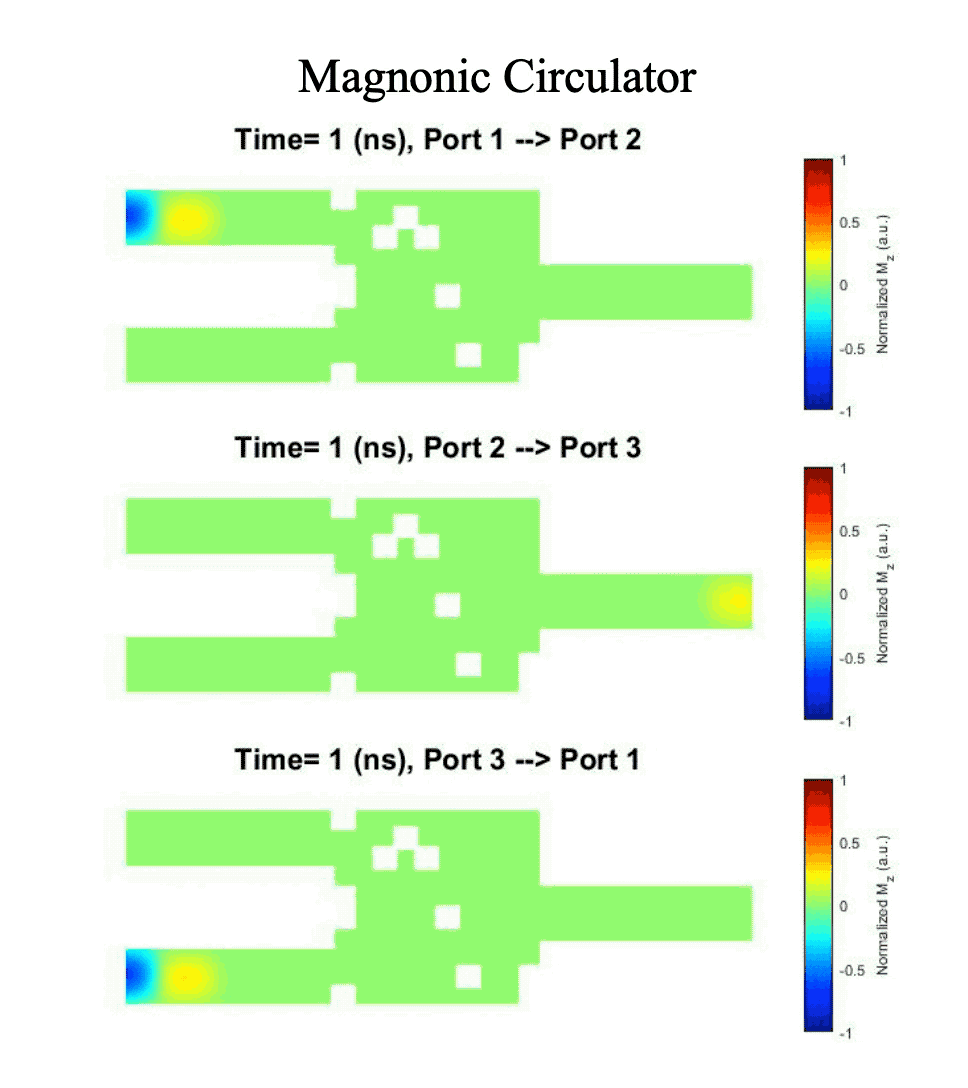

Supplement: Supplementary file 5 — Supplementary Move 4 - Magnonic circulator [file 41467_2021_22897_MOESM5_ESM.gif]
