# Supplementary material for: Inverse-design magnonic devices
Source: Nat Commun. 2021 May 11;12:2636. doi: 10.1038/s41467-021-22897-4 (PMC8113576; doi:10.1038/s41467-021-22897-4)
Supplement: Supplementary file 7 — Description of Additional Supplementary Files [file 41467_2021_22897_MOESM7_ESM.pdf]

Title: Supplementary Movie 1:
Description: Magnonic Demultiplexer

Title: Supplementary Movie 2:
Description: Magnonic Multiplexer

Title: Supplementary Movie 3:
Description: Magnonic Nonlinear Switch

Title: Supplementary Movie 4:
Description: Magnonic Circulator
